# The identification of high-performing antibodies for Charged multivesicular body protein 2b for use in Western Blot, immunoprecipitation and immunofluorescence

**DOI:** 10.12688/f1000research.139755.1

**Published:** 2023-07-25

**Authors:** Walaa Alshafie, Maryam Fotouhi, Riham Ayoubi, Irina Shlaifer, Kathleen Southern, Peter S. McPherson, Carl Laflamme, NeuroSGC/YCharOS/EDDU collaborative group

**Affiliations:** 1 Department of Neurology and Neurosurgery, Structural Genomics Consortium, The Montreal Neurological Institute, McGill University, Montreal, Québec, H3A 2B4, Canada; 2 The Neuro’s Early Drug Discovery Unit (EDDU), Structural Genomics Consortium, McGill University, Montreal, Québec, H3A 2B4, Canada

**Keywords:** Uniprot ID Q9UQN3, CHMP2B, Charged multivesicular body protein 2b, antibody characterization, antibody validation, Western Blot, immunoprecipitation, immunofluorescence

## Abstract

Charged multivesicular body protein 2B is a subunit of the endosomal sorting complex required for transport III (ESRCT-III), a complex implicated in the lysosomal degradation pathway and formation of multivesicular bodies. Mutations to the *CHMP2B* gene can result in abnormal protein aggregates in neurons and is therefore predicted to be associated in neurodegenerative diseases, including across the ALS-FTD spectrum. Through our standardized experimental protocol which compares read-outs in knockout cell lines and isogenic parental controls, this study aims to enhance the reproducibility of research on this target by characterizing eight commercial antibodies against charged multivesicular body protein 2b using Western Blot, immunoprecipitation, and immunofluorescence. We identified many high-performing antibodies and encourage readers to use this report as a guide to select the most appropriate antibody for their specific needs.

## Introduction

Charged multivesicular body protein 2B, encoded by the *CHMP2B* gene, is a core component of the endosomal sorting complex required for transport III (ESCTR-III) which plays a pivotal role in the biogenesis of multivesicular bodies (MVB) and is thus involved in endocytic trafficking of proteins. ^1^ MVB’s are late endosomes formed by scission of intraluminal vesicles from the limiting membrane of the endosome to then deliver cargo proteins to the lysosome, enabling degradation of membrane proteins. ^2^^,^^3^ As a subunit of the ESCRT-III complex, Charged multivesicular body protein 2 is essential to the pathway of lysosomal degradation.

Mutations to the *CHMP2B* gene have been predicted to be associated with amyotrophic lateral sclerosis (ALS) ^4^ and frontotemporal dementia (FTD). ^5^ Affected neurons having abnormal ubiquitin-positive protein deposits which can be attributed to dysfunctional lysosomal degradation. ^1^

*CHMP2B* mutations related to the ALS-FTD spectrum have advanced the understanding of the role endosomal-lysosomal and autophagic dysregulation play in neurodegeneration. ^1^ As the exact mechanisms remain unknown, the availability of high-quality Charged multivesicular body protein 2 antibodies would greatly facilitate mechanistic studies.

Here, we compared the performance of a range of commercially available antibodies for Charged multivesicular body protein 2b and identified high-performing antibodies for Western Blot, immunoprecipitation and immunofluorescence, enabling biochemical and cellular assessment of Charged multivesicular body protein 2 properties and function.

## Results and discussion

Our standard protocol involves comparing readouts from wild-type (WT) and knockout (KO) cells. ^6^^,^^7^ To identify a cell line that expresses adequate levels of Charged multivesicular body protein 2b protein to provide sufficient signal to noise, we examined public proteomics databases, namely PaxDB ^8^ and DepMap. ^9^ U2OS was identified as a suitable cell line and thus U2OS was modified with CRISPR/Cas9 to knockout the corresponding *CHMP2B* gene ( Table 1).

**Table 1.** Summary of the cell lines used.

Institution	Catalog number	RRID (Cellosaurus)	Cell line	Genotype
ATCC	HTB-96	CVCL_0042	U2OS	WT
Montreal Neurological Institute	-	CVCL_B6JX	U2OS	*CHMP2B* KO

**Figure 1.**
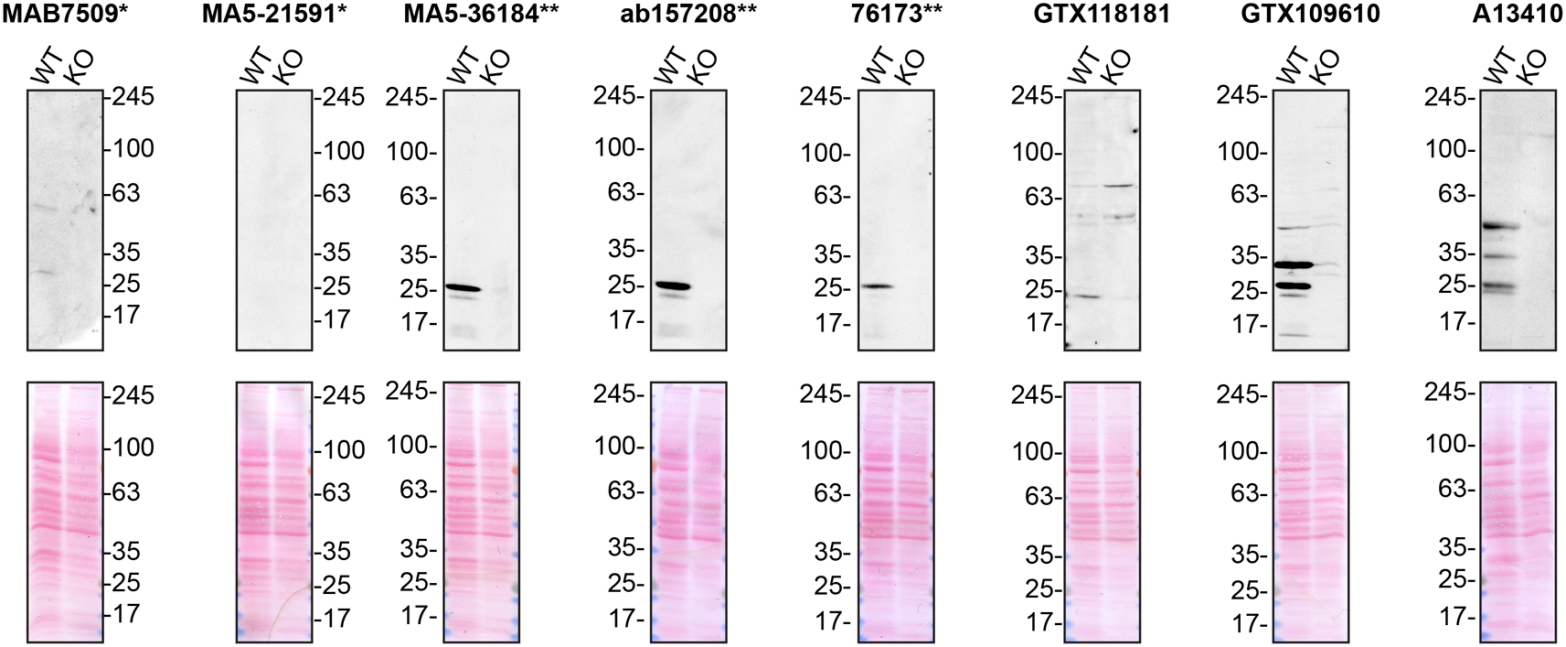
Charged multivesicular body protein 2b antibody screening by Western Blot. Lysates of U2OS (WT and *CHMP2B* KO) were prepared and 50 μg of protein were processed for Western Blot with the indicated Charged multivesicular body protein 2b antibodies. The Ponceau stained transfers of each blot are presented to show equal loading of WT and KO lysates and protein transfer efficiency from the acrylamide gels to the nitrocellulose membrane. Antibody dilutions were chosen according to the recommendations of the antibody supplier. When the concentration was not indicated by the supplier, which was the case for antibody MA5-21591*, the antibody was tested at 1/1000. Antibody dilution used: MAB7509* at 1/400; MA5-21591* at 1/1000; MA5-36184** at 1/500; ab157208** at 1/1000; 76173** at 1/1000; GTX118181 at 1/1000; GTX109610 at 1/1000; A13410 at 1/500. Predicted band size: 24 kDa. *Monoclonal antibody; **Recombinant antibody.

For Western Blot experiments, we resolved proteins from WT and *CHMP2B* KO cell extracts and probed them side-by-side with all antibodies in parallel ( Figure 1). ^7^^,^^10^^–^^19^

**Figure 2.**
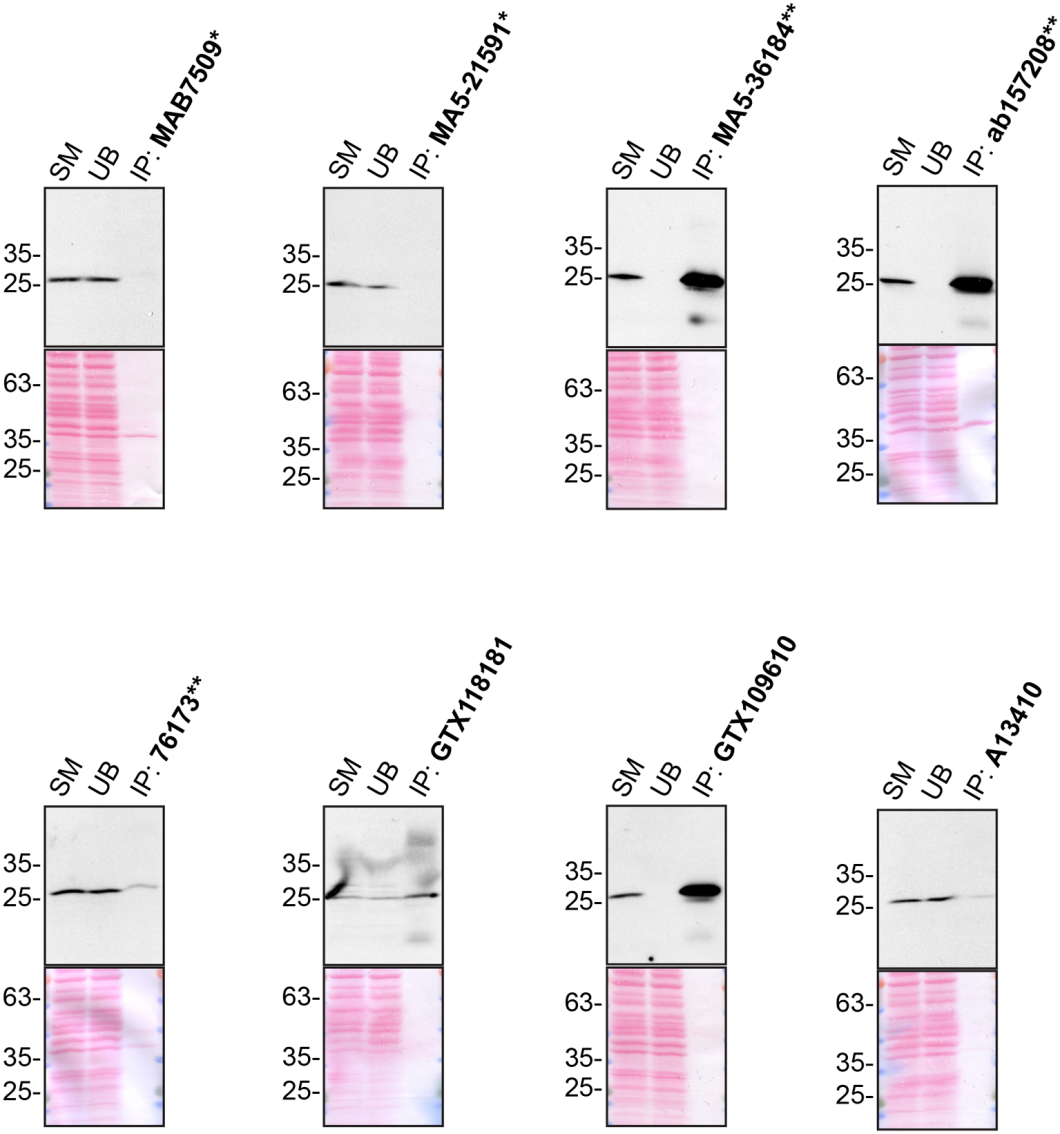
Charged multivesicular body protein 2b antibody screening by immunoprecipitation. U2OS lysates were prepared, and IP was performed using 1.0 μg of the indicated Charged multivesicular body protein 2b antibodies pre-coupled to Dynabeads protein G or protein A. Samples were washed and processed for Western Blot with the indicated Charged multivesicular body protein 2b antibody. For Western Blot, ab157208** was used at 1/2000. The Ponceau stained transfers of each blot are shown for similar reasons as in Figure 1. SM=10% starting material; UB=10% unbound fraction; IP=immunoprecipitated. *Monoclonal antibody; **Recombinant antibody.

For immunoprecipitation experiments, we used the antibodies to immunopurify Charged multivesicular body protein 2b from U2OS cell extracts. The performance of each antibody was evaluated by detecting the Charged multivesicular body protein 2b protein in extracts, in the immunodepleted extracts and in the immunoprecipitates ( Figure 2). ^7^^,^^10^^–^^19^

**Figure 3.**
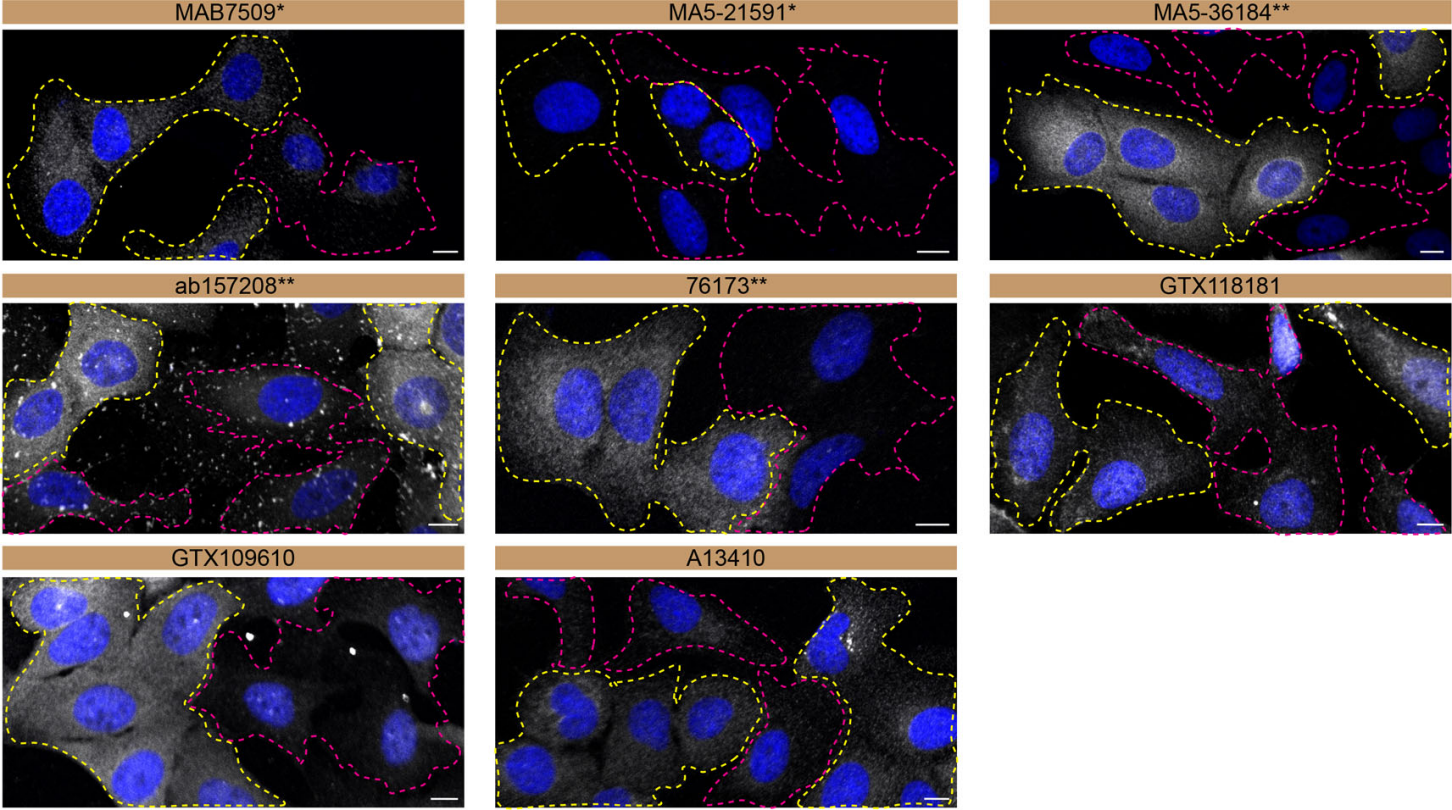
Charged multivesicular body protein 2b antibody screening by immunofluorescence. U2OS WT and *CHMP2B* KO cells were labelled with a green or a far-red fluorescent dye, respectively. WT and KO cells were mixed and plated to a 1:1 ratio on coverslips. Cells were stained with the indicated Charged multivesicular body protein 2b antibodies and with the corresponding Alexa-fluor 555 coupled secondary antibody including DAPI. Acquisition of the blue (nucleus-DAPI), green (WT), red (antibody staining) and far-red (KO) channels was performed. Representative images of the merged blue and red (grayscale) channels are shown. WT and KO cells are outlined with yellow and magenta dashed line, respectively. Antibody dilutions were chosen according to the recommendations of the antibody supplier. Exceptions were given to antibodies ab157208** and A13410, which were titrated to 1/1000 and 1/800, respectively, as the signals were too weak when following the suppliers' recommendations. When the concentration was not indicated by the supplier, which was the case for antibodies MA5-21791* and 76173*, we tested antibodies at 1/500. At this concentration, the signal from each antibody was in the range of detection of the microscope used. Antibody dilution used: MAB7509* at 1/500; MA5-21591* at 1/500; MA5-36184** at 1/1000; ab157208** at 1/100; 76173** at 1/500; GTX118181 at 1/500; GTX109610 at 1/1000; A13410 at 1/800. Bars=10 μm. *Monoclonal antibody; **Recombinant antibody.

For immunofluorescence, as described previously, antibodies were screened using a mosaic strategy. ^20^ In brief, we plated WT and KO cells together in the same well and imaged both cell types in the same field of view to reduce staining, imaging and image analysis bias ( Figure 3).

In conclusion, we have screened Charged multivesicular body protein 2b commercial antibodies by Western Blot, immunoprecipitation and immunofluorescence and identified several high-quality antibodies under our standardized experimental conditions. The underlying data can be found on Zenodo. ^21^^,^^22^

## Methods

### Antibodies

All Charged multivesicular body protein 2b antibodies are listed in Table 2, together with their corresponding Research Resource Identifiers, or RRID, to ensure the antibodies are cited properly. ^23^ Peroxidase-conjugated goat anti-rabbit and anti-mouse antibodies are from Thermo Fisher Scientific (cat. number 65-6120 and 62-6520). Alexa-555-conjugated goat anti-rabbit and anti-mouse secondary antibodies are from Thermo Fisher Scientific (cat. number A21429 and A21424).

**Table 2.** Summary of the Charged multivesicular body protein 2b antibodies tested.

Company	Catalog number	Lot number	RRID (Antibody Registry)	Clonality	Clone ID	Host	Concentration (μg/μl)	Vendors recommended applications
Bio-Techne	MAB7509 *	CHEB0112101	AB_2885148	monoclonal	791521	mouse	0.50	Wb
Thermo Fisher Scientific	MA5-21591 *	WA3152391	AB_2576481	monoclonal	2H6-1E6	mouse	0.50	Other application
Thermo Fisher Scientific	MA5-36184 **	VL3152619	AB_2890433	recombinant-mono	JE54-35	rabbit	1.00	Wb, IF
Abcam	ab157208 **	GR117930-3	AB_2885096	recombinant-mono	EPR10807(B)	rabbit	0.13	Wb, IP, IF
Cell Signaling Technology	76173 **	1	AB_2799880	recombinant-mono	D4G3K	rabbit	not provided	Wb, IP
GeneTex	GTX118181	40625	AB_11174469	polyclonal	-	rabbit	0.59	Wb, IF
GeneTex	GTX109610	40681	AB_11163162	polyclonal	-	rabbit	1.00	Wb, IF
ABclonal	A13410	13540101	AB_2760272	polyclonal	-	rabbit	0.85	Wb, IF

Wb=Western blot; IF=immunofluorescence; IP=immunoprecipitation.

* Monoclonal antibody.

** Recombinant antibody.

### CRISPR/Cas9 genome editing

Cell lines used are listed in Table 1. U2OS *CHMP2B* KO clone was generated with low passage cells using an open-access protocol available on Zenodo.org. The sequence of the guide RNA is the following: CCAAACAACUUGUGCAUCUA.

### Cell culture

Both U2OS WT and *CHMP2B* KO cell lines used are listed in Table 1, together with their corresponding RRID, to ensure the cell lines are cited properly. ^24^ Cells were cultured in DMEM high glucose (GE Healthcare cat. number SH30081.01) containing 10% fetal bovine serum (Wisent, cat. number 080450), 2 mM L-glutamate (Wisent cat. number 609065, 100 IU penicillin and 100 μg/mL streptomycin (Wisent cat. number 450201).

### Antibody screening by Western Blot

Western Blots were performed as described in our standard operating procedure. ^25^ U2OS WT and *CHMP2B* KO were collected in RIPA buffer (25 mM Tris-HCl pH 7.6, 150 mM NaCl, 1% NP-40, 1% sodium deoxycholate, 0.1% SDS) (Thermo Fisher Scientific, cat. number 89901) supplemented with 1× protease inhibitor cocktail mix (MilliporeSigma, cat. number P8340). Lysates were sonicated briefly and incubated for 30 min on ice. Lysates were spun at ~110,000 × g for 15 min at 4°C and equal protein aliquots of the supernatants were analyzed by SDS-PAGE and Western Blot. BLUelf prestained protein ladder (GeneDireX, cat. number PM008-0500) was used.

Western Blots were performed with large 4-20% polyacrylamide gels and transferred on nitrocellulose membranes. Proteins on the blots were visualized with Ponceau S staining (Thermo Fisher Scientific, cat. number BP103-10) which is scanned to show together with individual Western Blot. Blots were blocked with 5% milk for 1 hr, and antibodies were incubated overnight at 4°C with 5% bovine serum albumin (BSA) (Wisent, cat. number 800-095) in TBS with 0.1% Tween 20 (TBST) (Cell Signalling Technology, cat. number 9997). Following three washes with TBST, the peroxidase conjugated secondary antibody was incubated at a dilution of ~0.2 μg/mL in TBST with 5% milk for 1 hr at room temperature followed by three washes with TBST. Membranes were incubated with Pierce ECL (Thermo Fisher Scientific, cat. number 32106) prior to detection with the HyBlot CL autoradiography films (Denville, cat. number 1159T41).

### Antibody screening by immunoprecipitation

Immunoprecipitation was performed as described in our standard operating procedure. ^26^ Antibody-bead conjugates were prepared by adding 1 μg or 2 μL of antibody at an unknown concentration to 500 μL of Pierce IP Lysis Buffer (Thermo Fisher Scientific, cat. number 87788) in a 1.5 mL microcentrifuge tube, together with 30 μL of Dynabeads protein A - (for rabbit antibodies) or protein G - (for mouse antibodies) (Thermo Fisher Scientific, cat. number 10002D and 10004D, respectively). Pierce IP Lysis Buffer was supplemented with the Halt Protease Inhibitor Cocktail 100X (Thermo Fisher Scientific, cat. number 78446) at a final concentration of 1×. Tubes were rocked for ~2 hrs at 4°C followed by several washes to remove unbound antibodies.

U2OS WT were collected in Pierce IP buffer (25 mM Tris-HCl pH 7.4, 150 mM NaCl, 1 mM EDTA, 1% NP-40 and 5% glycerol) supplemented with protease inhibitor. Lysates were rocked for 30 min at 4°C and spun at 110,000 × g for 15 min at 4°C. One mL aliquots at 1.0 mg/mL of lysate were incubated with an antibody-bead conjugate for ~2 hours at 4°C. The unbound fractions were collected, and beads were subsequently washed three times with 1.0 mL of IP lysis buffer and processed for SDS-PAGE and Western Blot on a 4-20% polyacrylamide gels. Prot-A:HRP (MilliporeSigma, cat. number P8651) was used as a secondary detection system at a dilution of 0.4 μg/mL for an experiment where a rabbit antibody was used for both immunoprecipitation and its corresponding immunoblot.

### Antibody screening by immunofluorescence

Immunofluorescence was performed as described in our standard operating procedure. ^7^^,^^10^^–^^20^ U2OS WT and *CHMP2B* KO were labelled with a green and a far-red fluorescence dye, respectively (Thermo Fisher Scientific, cat. number C2925 and C34565). The nuclei were labelled with DAPI (Thermo Fisher Scientific, cat. number D3571) fluorescent stain. WT and KO cells were plated on glass coverslips as a mosaic and incubated for 24 hrs in a cell culture incubator at 37 ^o^C, 5% CO. Cells were fixed in 4% paraformaldehyde (PFA) (Beantown chemical, cat. number 140770-10 ml) in phosphate buffered saline (PBS) (Wisent, cat. number 311-010-CL). Cells were permeabilized in PBS with 0.1% Triton X-100 (Thermo Fisher Scientific, cat. number BP151-500) for 10 min at room temperature and blocked with PBS containing 5% BSA, 5% goat serum (Gibco, cat. number 16210-064) and 0.01% Triton X-100 for 30 min at room temperature. Cells were incubated with IF buffer (PBS, 5% BSA, 0.01% Triton X-100) containing the primary Charged multivesicular body protein 2b antibodies overnight at 4°C. Cells were then washed 3 × 10 min with IF buffer and incubated with corresponding Alexa Fluor 555-conjugated secondary antibodies in IF buffer at a dilution of 1.0 μg/mL for 1 hr at room temperature with DAPI. Cells were washed 3 × 10 min with IF buffer and once with PBS. Coverslips were mounted on a microscopic slide using fluorescence mounting media (DAKO).

Imaging was performed using a Zeiss LSM 880 laser scanning confocal microscope equipped with a Plan-Apo 63× oil objective (NA=1.40). Analysis was done using the Zen navigation software (Zeiss). All cell images represent a single focal plane. Figures were assembled with Adobe Photoshop (version 24.1.2) to adjust contrast then assembled with Adobe Illustrator (version 27.3.1).

## Data Availability

Zenodo: Antibody Characterization Report for Charged multivesicular body protein 2b, https://doi.org/10.5281/zenodo.6370501. ^21^ Zenodo: Dataset for the Charged multivesicular body protein 2b antibody screening study, https://doi.org/10.5281/zenodo.8139356. ^22^ Data are available under the terms of the Creative Commons Attribution 4.0 International license (CC-BY 4.0).
