# Supplementary material for: The identification of high-performing antibodies for Charged multivesicular body protein 2b for use in Western Blot, immunoprecipitation and immunofluorescence
Source: F1000Res. 2023 Jul 25;12:884. [Version 1] doi: 10.12688/f1000research.139755.1 (PMC10448144; doi:10.12688/f1000research.139755.1)
Supplement: Peer review history

# Peer review: Reviewer response for version 1

**DOI:** 10.5256/f1000research.153058.r196057

**Published:** 2023-08-23

**Authors:** Xiao-Xin Yan

**Affiliations:** 1 Department of Anatomy and Neurobiology, Central South University Xiangya School of Medicine, Changsha, Hunan, China

This manuscript reports the characterization of *CHMP2B* antibodies for the use in Western Blot, immunoprecipitation and immunofluorescence. Good *CHMP2B *antibodies would be very useful for study of not only ALS-FTD disorders as the authors indicated, but also for other neurodegenerative diseases or conditions, such as aging and AD-related formation of granulovacuolar degeneration (GVD) (Jiang *et al.*, 2022 ^1^). The utility of the antibodies in immunoprecipitation is particularly informative for research into potential interplay between *CHMP2B *and other molecular partners in the neuroscience field. I would appreciate if the authors could extend some more relavence either in the introduction or discussion on *CHMP2B in relevance to GVD formation or protein sorting in neurodegeneratrive disorders.*

Are sufficient details of methods and materials provided to allow replication by others?

Yes

Is the rationale for creating the dataset(s) clearly described?

Yes

Are the datasets clearly presented in a useable and accessible format?

Yes

Are the protocols appropriate and is the work technically sound?

Yes

Reviewer Expertise:

Neurodevelopment, brain aging, Alzheimer's disease

I confirm that I have read this submission and believe that I have an appropriate level of expertise to confirm that it is of an acceptable scientific standard.

## References

[1] : Intraneuronal sortilin aggregation relative to granulovacuolar degeneration, tau pathogenesis and sorfra plaque formation in human hippocampal formation. *Front Aging Neurosci* .2022;14: 10.3389/fnagi.2022.926904 926904 10.3389/fnagi.2022.926904 35978952PMC9376392

# Peer review: Reviewer response for version 1

**DOI:** 10.5256/f1000research.153058.r190521

**Published:** 2023-08-08

**Authors:** Emma L. Clayton

**Affiliations:** 1 UK Dementia Research Institute, King's College London, London, UK

In line with the remit of the YCharOS (antibody characterisation through open science) initiative which seeks to characterise commercially available antibodies for every human protein, Alshafie *et al.* have compared the ability of a range of commercially available antibodies to detect CHMP2B by western blotting and immunofluorescence. The model used for these validation experiments (U2OS) was selected as a line known to express adequate levels of CHMP2B to provide sufficient signal to noise. Antibody validation was complemented through use of CRISPR/Cas9 knockout to generate CHMP2B deficient control lines.

 This work is extremely helpful for researchers looking to source the best commercially available antibody to investigate their protein of interest, in particular the use of KO lines as a control to fully validate the specificity of the bands seen on the western blot images is powerful. The inclusion of the ponceau membranes to show protein transfer, and the inclusion of the whole length of the blot (to show non-specific bands) is a thorough and informative presentation of the antibody validation.

 For the immunofluorescence data, the mosaic plating of the cells to allow the direct comparison of staining in adjacent cells allows for direct comparison and removes any variability due to differences in the staining protocol between conditions. This makes the comparisons between cells extremely powerful within images.

 I could suggest two additions to the paper that would be interesting for researchers looking at CHMP2B and would add to the general usefulness of this antibody insight for the research community.

 CHMP2B is generally cytosolic, and is temporarily recruited to the ESCRT-III complex to facilitate membrane invagination prior to VPS4 recruitment which then allows for membrane scission and ESCRT-III disassembly. When VPS4 is not fusion competent, CHMP2B is held in punctate structures in the cell ^1^. In the immunofluorescence images in this article, the CHMP2B looks highly cytosolic as expected, however the adjacent KO cells don’t look particularly different to the WT cells. It is hard to see what is specific staining over background noise. To make this immunofluorescence antibody validation useful for the research community, inducing the punctate localisation of CHMP2B would be useful, in order to show true antibody specificity.

 I would also suggest adding the recognition sequences for the antibodies. This would be helpful in the case of mutations in CHMP2B which are associated with frontotemporal dementia and amyotrophic lateral sclerosis ^2^. In particular, the mutation of CHMP2B associated with a familial form of frontotemporal dementia results in a C terminally truncated form of the protein ^3^. If the antibody recognition sequence resides within the truncated portion of the protein (as we have unfortunately encountered in the past with CHMP2B antibodies), then the usefulness of an otherwise “good” CHMP2B antibody is compromised. Thus this information would be helpful for the research community interested in CHMP2B in neurodegenerative disease.

 In summary however, this paper is a powerful resource for any researchers looking to work with wildtype CHMP2B. The detailed methods (with antibody dilutions and table of research resource identifiers) and in particular the powerful direct comparisons of WT and KO by western blotting provide high confidence in commercial antibody choice for a researcher looking to select an antibody for CHMP2B research.

Are sufficient details of methods and materials provided to allow replication by others?

Yes

Is the rationale for creating the dataset(s) clearly described?

Yes

Are the datasets clearly presented in a useable and accessible format?

Yes

Are the protocols appropriate and is the work technically sound?

Yes

Reviewer Expertise:

Neurodegeneration, Membrane trafficking, Synaptopathy

I confirm that I have read this submission and believe that I have an appropriate level of expertise to confirm that it is of an acceptable scientific standard.

## References

[1] : Frontotemporal dementia causative CHMP2B impairs neuronal endolysosomal traffic-rescue byTMEM106B knockdown. *Brain* .2018;141(12) : 10.1093/brain/awy284 3428-3442 10.1093/brain/awy284 30496365PMC6262218

[2] : Lessons learned from CHMP2B, implications for frontotemporal dementia and amyotrophic lateral sclerosis. *Neurobiology of Disease* .2021;147: 10.1016/j.nbd.2020.105144 10.1016/j.nbd.2020.105144 33144171

[3] : Mutations in the endosomal ESCRTIII-complex subunit CHMP2B in frontotemporal dementia. *Nature Genetics* .2005;37(8) : 10.1038/ng1609 806-808 10.1038/ng1609 16041373
